# Twenty Key Challenges in Environmental and Resource Economics

**DOI:** 10.1007/s10640-020-00516-y

**Published:** 2020-10-16

**Authors:** Lucas Bretschger, Karen Pittel

**Affiliations:** 1 CER-ETH Centre of Economic Research at ETH Zurich, ZUE F7, 8092 Zurich, Switzerland; 2 ifo Center for Energy, Climate and Resources, ifo Institute and LMU Munich, Munich, Germany

**Keywords:** Environmental and resource economics, Survey, Key research topics, Q00, Q2, Q3, Q5

## Abstract

Economic and ecological systems are closely interlinked at a global and a regional level, offering a broad variety of important research topics in environmental and resource economics. The successful identification of key challenges for current and future research supports development of novel theories, empirical applications, and appropriate policy designs. It allows establishing a future-oriented research agenda whose ultimate goal is an efficient, equitable, and sustainable use of natural resources. Based on a normative foundation, the paper aims to identify fundamental topics, current trends, and major research gaps to motivate further development of academic work in the field.

## Introduction

### Research Frontier

The research agenda in environmental and resource economics has always been very broad and dynamic, reflecting the ways our economies interact with the natural environment. While in classical economics of the eighteenth century the factor land played a dominant role, the effects of pollution externalities, resource scarcities, ecosystem services, and sustainability became important in subsequent time periods. These issues have triggered different waves of research with very prominent results, specifically on optimal policies in the presence of externalities (Pigou 1920), optimal extraction of non-renewable resources (Hotelling 1931), optimal capital accumulation in the presence of resource scarcities (Dasgupta and Heal 1974), and sustainable development (Hartwick 1977; Pearce et al. 1994). Of course, the list of topics has already been very diverse in the past but has increasingly become so with recent global environmental problems challenging the functioning of a world economy which is growing at a high rate and heavily relies on an international division of labour and trade.

In the past, new research challenges emerged and manifested in different ways: Some topical fields became increasingly relevant due to new technological developments, new ecological or societal challenges or new political agendas. Others arose in fields that were already well researched but rose in importance. Not all challenges were of a topical nature. In some fields, we found our methodological tool-kit not equipped to deal with new problems or in need of extension to find new (and better) answers to old questions. At the same time, it has become increasingly clear that we have to reach out to other disciplines to meet new and often immense challenges. In environmental economics it is key to seek a good balance between disciplinary excellence, interdisciplinary collaboration, and political impact.

Environmental and resource economics is a dynamic field, in which new key topics emerge frequently. So, while the topical and methodological challenges that the paper identifies will be important for some time to come, they will and should also be subject to further development over the next years and decades. The paper aims to identify and address the variety of new complex problems generated by humans when they exploit natural resources and the environment. We specifically identify *Twenty Challenges* that we feel will be important for environmental and resource economists to address. We are aware that such a list will never be unanimously agreed upon and we do not even lay claim on the list being complete; the next section provides a background to the compilation of the list. Nevertheless, we feel it to be important to (at best) point researchers in directions important to work in in the future or (at least) to launch a new—controversial but productive—discussion on the development of our field. In any case, the paper should support the profession to operate at the research frontier generating novel theories, empirical designs, and workable policies. But, before we turn to the *Twenty Challenges*, we aim to motivate the framing of research in our field—past, present and future.

### Identification of Research Challenges

To provide a normative foundation for our research agenda we characterize our underlying assumptions and generalized views on the nature of research in the field. This set of basic assumptions motivates the criteria of importance, activeness, and distinction of the selected topics as well as our choices with respect to design, methodology and research methods. Identifying the relevant issues, i.e. the mere choice of what to study in environmental economics imposes specific values on the subjects. In our view, the guiding principle in the normative framework is that environmental economics differs from general economics by its ontology, i.e. the system of belief that reflects the interpretation of what constitutes an important fact. It is a deep and serious concern about the state of the natural environment that drives the economic analysis of ecological processes. Nature is not simply part of the economic system but a different system with its own very complex regularities and dynamics; ecosystem values are not reducible to market exchange values. The task to integrate the ecological and economic systems to a holistic framework in an appropriate manner and to derive valid guidelines for the economy under the restrictions imposed by the environment lies at the heart of our research. Central parts of the ontology are the valuation of ecosystems, the increasing scarcities in natural resources and sinks, the effects of environmental externalities, the long-term orientation of planning, an important role of uncertainty, and the existence of irreversible processes. The anthropocentric view and the use of utilitarianism do not imply that individuals are purely self-centered and narrowly selfish. It highlights the indistinguishable role of human decision making for the future of the planet and aims at decision making that cares for efficiency, equity, and posterity. Based on a broad utilitarian setup, growth is not valued in terms of material consumption but in terms of wellbeing, which includes elements like social preferences, work-life balance, appreciation of nature etc. Posterity reflects our care for future generations, whose welfare should not be harmed by the activities of current generations. Fundamental changes of the economy e.g. the phase-out of fossil fuels, includes policy-induced decrease of activities, a role for technology, substitutability in production and consumption, a decoupling from natural resource use, and internalizing cost to correct market failures. Substantive transitions are very difficult to implement, as important lock-in mechanisms such as habit persistence, built infrastructure, and supporting policies such as subsidies stabilize current practices. To achieve a change of mindset in politics to achieve a transition to a green economy is a difficult task. A fundamental systems change, as discussed by many these days, is undoubtedly much more complex to accomplish; its impacts are uncertain and may delay the necessary steps which are important to rapidly improve the state of our ecosystems.

We acknowledge that one can always challenge an ontological position because it reflects ethical principles. In our research agenda there is no external reality, independent of what we may think or understand it to be. We reduce economic and ecological complexity through our personal system of belief to design our preferred map, which by definition is not the territory. In his survey of ecological research issues for the economists, Ehrlich (2008) refers to his ”own mental meta-analysis” to motivate his choices and to alert us to the importance of research on big issues like the meaning of life, mortality, and death. At the same time, he acknowledges that the emergence of pervasive new environmental problems, such as climate change and biodiversity loss, requires to flexibly adjust research programs to societal demand. Adjustments of the agenda may also be supply driven, when new methods allow for more effective engagement with important issues like risk and uncertainty or assessment of empirical regularities with superior estimation methods.

### Forming a Research Agenda

Environmental economics is closely linked to general economics in its epistemology, i.e. the validity, scope and methods of acquiring knowledge by using models, distinguishing between positive and normative models, and testing hypotheses with empirical methods and experiments. An important cornerstone for economic research has always been the analysis of economic efficiency. Since the early days of environmental economics research, this has also held for our field whether it concerned the efficiency in the use of natural resources or the design of policies. Although research in our field has become much more interdisciplinary and policy-oriented, this still constitutes common ground. It is still a prime duty of the economist to point at the potentially vast allocative inefficiencies of the use of natural resources in pure market economies. Efficiency is a necessary condition for optimal states of the economic-ecological system and the foundation for policies maximizing social welfare.

The pursuit of optimality has to be complemented by a requirement to take care of equity and posterity enabling sustainability of development. In this long-run perspective, economics has to highlight the substitution effect as a powerful mechanism establishing consistency between humanity and its natural environment. Substitution comes in many guises, e.g. as substitution between clean and dirty production, renewable and exhaustible resources, extractive and conservationist attitude, pollution intensive and extensive consumption, etc. This dynamic analysis is crucial in many respects. It has recently been included at all levels of research in the fields. The same holds for the issue of risk and uncertainty, a pervasive topic when dealing with the environment.

In many cases, there has been a significant discrepancy between the theoretical derivation of social optima in academia and the attempts to foster their implementation under realistic policy conditions. As a consequence, policies dealing with environmental issues have been of very different quality and effectiveness. The reduction of acid rains, the protection of the ozone layer, and cutbacks of particulate matter emissions in many world regions were among the prominent successes. Global warming, extraction of rare earth elements, and loss of biodiversity are not yet addressed in a comprehensive manner. Political resistance against the protection of nature often refers to the economic costs of policies, including the concerns of growth reduction, employment loss, and adverse effect on income distribution. The lack of success in many policy areas has led to reformulation and extension of the research agenda. In the future, research should focus more on strengthening the links between theory and policy.

Our selection of the *Twenty Challenges* is also based on the potential of research in these areas to contribute and leverage social welfare and sustainable development. We specifically look for areas that are either inherently new to the research agenda in environmental and resource economics or in which research stagnates. We present the challenges in a specific order and like to highlight the links between them before we enter into the details. The aim of net zero carbon emission by the mid of the century dominates current policy debates and unites basically all important elements of our discipline; it thus constitutes a good starting point. Decarbonization necessarily involves a deep understanding of systems dynamics and of risk and resilience, which are presented next. An important and not sufficiently addressed research issue is the emergence of disruptive development during a substantive transition, the next challenge for our research. Extending the scope, we then address human and government behaviour. In the context of environmental policy, the popular and sometimes underrated request of an equitable use of the environment has emerged as a dominant topic, a next issue for further research. As natural capital involves many more elements than the climate, biodiversity and general ecosystem services are included in the sequence. Broadening the scope to the big problems of human behaviour with natural resources we then turn to political conflicts, population development and conflicting land use. Shifting the focus on induced movements of the labour force we go on by dealing with environmental migration and urbanization. These affect welfare of the individuals in a major way, like health and the epidemiological environment as a next research challenge. In terms of the reorganization of the transition to a green economy we highlight the central role of finance and the implementation of new measures in the dominant energy sector. The final three research challenges are motivated by advances in the methodology. Big data and machine learning offer new perspectives in sustainability research, refined methods and increasing experience improve our simulation models and structural assessment modelling, which forms the last three challenges of our list.

### Links to Current Research

In order to put our agenda into a broader perspective and to concretize the selected challenges, we believe it is important to show the relationship between our research agenda and the priorities in current literature and policy debates. We have considered three main links. First, we conducted a quantitative and qualitative literature review and analyzed current research as presented at international conferences (World Conference of Environmental and Resource Economics in 2018, the SURED conference in 2018, Meetings of the American, European, and Asian Associations of Environmental and Resource Economics in 2019). The aim of this analysis was to see where our profession moves and which of the currently hotly debated topics offers a high potential for future research. Second, we took the discussions in interdisciplinary research fora into consideration to identify further fields that are of high importance for future resource use, sustainable development and environmental outcomes but have so far not been adequately addressed from an economics perspective. Information on this research was gained through interdisciplinary research initiatives (for example The Belmont Forum, Future Earth and National Research Funding Activities). Involvement in interdisciplinary and globally oriented research councils provided further access to the discussions in other disciplines. Third, we draw conclusions from current policies and news as well as our involvement in the policy arena. The authors are involved in a number of institutionalized policy-oriented activities on the regional, national and international level (Regional Climate Councils, National Climate Policy Platforms as well as the UN climate negotiations).

The paper relates to similar contributions in recent literature. Based on citation data Auffhammer (2009) identifies important topics and scholars and provides a brief historical overview of the discipline from exhaustible and renewable resources to sustainability, pollution control, development, international trade, climate change, international agreements, and non-market valuation. Polyakov et al. (2018) analyze authorship patterns using text analysis for classification of articles in *Environmental and Resource Economics.* Based on 1630 articles published in the Journal from 1991 to 2015 they document the importance of applied and policy-oriented content in the field. They identify non-market valuation, recreation and amenity, and conservation, as popular topics and growing when measured by both number of articles and citations. Costanza et al. (2016) investigate the most influential publications of *Ecological Economics* in terms of citation counts both within the journal itself and elsewhere. Important topics turn out to be social aspects of environmental economics and policy, valuation of environmental policy, governance, technical change, happiness and poverty, and ecosystem services. A contemporary analysis of how research issues have developed in the *Journal of Environmental Economics and Management* in the time of its existence is provided by Kubea et al. (2018). These authors show that the sample of topics has broadened from the core issues of non-market valuation, cost-benefit analysis, natural resource economics, and environmental policy instruments to a more diversified array of research areas, with climate change and energy issues finding their way into the journal. In addition, increasing methodological plurality becomes apparent. They conclude that energy, development, and health are on the rise and that natural resources, instrument choice, and non-market valuation will endure; multidisciplinary work will be increasingly important. An excellent survey on research in the central field of sustainable development is provided in Polasky et al. (2019), which explicitly shows where the collaboration between economists and the other disciplines is currently insufficient and how it should be intensified in the future.

Regarding the literature that we connect our *Twenty Challenges* to, we naturally face the problem that some challenges have so far not been addressed adequately in the (economics) literature. In these cases we also reference papers from other disciplines. We, however, also take basic literature and recent research in environmental and resource economics into account. As we often deal with emerging topics, we cite some of this work even when not yet published. In other cases, where future research can build on or learn from past research, we also go back in time and reference older papers. Ultimately, neither our list of challenges nor the literature we base our analysis on will be satisfying to everybody. Our selection cannot be comprehensive and does not claim to be. But the specific task to identify future-oriented topics ultimately lasts on a subjective individual assessment of the authors. Nevertheless, hopefully it imparts impulses for future research in the different subfields of environmental and resource economics.

## Twenty Challenges

The ordering of the following challenges should not be understood to perfectly reflect their individual importance (beyond what we explained in the previous sections). Also, many of the fields discussed are inherently related, creating some unavoidable overlap. We feel that efforts to bring the challenges into some complete ’natural order’ are not only doomed to fail but also would not do them justice as they relate to very different areas and can/should not be weighed against each other. Also, attempting to show their interrelations would result in a 20-by-20 matrix that would not provide more clarity.

1. **Deep decarbonization and climate neutrality** To limit global warming to a maximum of 1.5 degrees Celsius, a state of net zero greenhouse gas emissions—i.e. climate neutrality—should be reached by the mid of the century (IPCC 2018). The directly following and unprecedented challenge is to decarbonize the global economy in very a narrow time window (Hainsch et al. 2018). This holds especially as the threshold for 1.5 degrees is expected to be passed around 2040 (IPCC 2018). Countries must increase their NDC ambitions of the Paris Agreement more than fivefold to achieve the 1.5 degree goal (UN - United Nations 2019). The time window for necessary decisions is closing fast. Infrastructure that is installed today often has a life span that reaches until and beyond 2050. Decisions on investments today therefore affect the ability to reach climate targets not only in 2030 but also 2050 and beyond. And while the necessity of reaching net zero emissions by mid century is reflected by, e.g., the European Commission’ Green Deal, much uncertainty remains regarding its implementation. This holds to an even larger extent with respect to other countries and regions. The fundamental challenge is to better understand economically viable deep decarbonization paths and then to implement incentives for input substitution, technology development, and structural change. More specifically, the vision of these policies has to be long-term and reach beyond phasing out coal and increasing energy efficiency. However, despite recent research efforts in climate economics, many issues around decarbonization, negative emissions and economic development are still controversial or insufficiently understood by economists. Specifically, industry applications for which alternative technologies are not available yet as well as agricultural emissions will have to be addressed. Also, the later greenhouse gas emissions start to fall, the faster their decline will have to ultimately be in order not to overshoot temperature targets (Agliardi and Xepapadeas 2018), leading to an increased need for negative emissions. However, potential trade-offs and synergies in the use of land for negative emission technologies, food production and biodiversity are still underresearched. Identifying technologies today that are the most promising in the very long run is subject to high uncertainty. Yet, while investing too early might be costly, delaying investment might cost even more or might lead to a weakening of future climate targets (Gerlagh and Michielsen 2015). Also, transition processes may involve strong scale effects implying nonlinear development of abatement cost. Once certain thresholds are reached, lower abatement cost or even disruptive development completely altering the production process could emerge in a later phase of decarbonization. Given the dramatic increase needed in mitigation efforts to reach the 1.5 or even 2 degree target, more attention also has to be devoted to the question of adaptation. Until today, the focus of research as well as policy has been primarily on mitigation rather than adaptation, partially because of expected substitution effects between mitigation and adaptation and partially because adaptation was taken to be automatic (Fankhauser 2017). However, as Fankhauser lays out “knowledge gaps, behavioral barriers, and market failures that hold back effective adaptation and require policy intervention”. All of these topics present a wide scope for substantial further research.
2. **Dynamics of the economic-ecological system ** Depletion of exhaustible resources, harvesting of renewable resources, recycling of raw materials, and accumulation of pollution stocks require basic societal decisions which are of an inherently dynamic nature. Whether the world society will be able to enjoy constant or increasing living standards under such dynamic natural constraints depends on another dynamic process, which is the accumulation of man-made capital. To derive the precise laws of motion in all the stock variables is challenging because general solutions of dynamic systems with several states are usually hard to obtain. An adequate procedure to obtain closed-form solutions may be to link several stocks in a reasonable way, e.g. when simultaneously dealing with resource, pollution, and capital stocks (Peretto 2017; Bretschger 2017b). The specific challenge is then to find the best possible economic justification to motivate the links. One may also focus on a few stocks which are considered the main drivers of economic development and sustainable growth on a global scale (Marin and Vona 2019; Borissov et al. 2019). When resorting to numerical simulation methods it is a main challenge to provide basic economic results which are sufficiently robust and supported by ample economic intuition. Social-ecological systems are increasingly understood as complex adaptive systems. Essential features of these systems - such as nonlinear feedbacks, strategic interactions, individual and spatial heterogeneity, and varying time scales—pose another set of substantial challenges for modeling in a dynamic framework. A main challenge is the characterization and selection of dynamic paths with multiple equilibria and the overall tractablility of the models, given the diversity of interlinkages and nonlinear relationships. The complexity of economic-ecological systems lead to a main challenge for designing effective policies is taking account of network effects, strategic interaction, sectoral change, path dependencies, varying time lags, and nonlinear feedbacks have to be considered as well as different regional and temporal scales, interdependencies between ecosystems, institutional restrictions and distributional implications (see, e.g., Engel et al. 2008; Levin et al. 2013; Vatn 2010). Optimal policies should also acknowledge the balance between the preservation of the ecology and the development of the economy especially for countries growing out of poverty. Setting a price for ecosystem services and natural capital via policy is important for preventing innovation incentives from being skewed against maintaining natural capital and ecosystem services.
3. **Risk, uncertainty, and resilience ** The vast majority of contributions in environmental economics use models with a purely deterministic structure. However, large negative environmental events require a completely different framework, which poses specific challenges for modelling. Heatwaves, floods, droughts, and hurricanes are shocks that are very uncertain, arriving at irregular times and with varying intensity. Also, risk and uncertainty about socio-economic impacts and technological development affect the optimal design of policies (see, e.g., Jensen and Traeger 2014). Moreover, uncertainty changes the political economy of climate policy and, finally, regulatory and policy uncertainty might create obstacles to reach climate targets through, for example, distortions of investment decisions (Pommeret and Schubert 2018; Bretschger and Soretz 2018). Stern (2016) argued forcefully that climate economics research needs to better integrate risk and uncertainty. Bigger disasters or so-called ”tipping points” such as the melting of the Greenland ice sheet, the collapse of Atlantic thermohaline circulation, and the dieback of Amazon rainforest involve an even higher level of uncertainty (Lenton and Ciscar 2013) with implications for optimal policy design and capital accumulation (Van der Ploeg and de Zeeuw 2018). Understanding the implications of tipping points is further complicated as the different tipping points are not independent of each other (Cai et al. 2016). The Economy and the Earth system both form non-deterministic systems; combining the two in an overarching framework and adding institutions for decision making multiplies the degree of complexity for adequate modelling and methods (Athanassoglou and Xepapadeas 2012). It is thus a main challenge for further research to provide analytic foundations and policy rules for rational societal decision-making under the conditions of risk and uncertainty up to deep uncertainty (Brock and Xepapadeas 1903; Baumgärtner and Engler 2018). Future work on policy design under deep uncertainty can build on a wide range of literature ranging from the assessment of the precautionary principle in this context to the fundamental contributions by Hansen and Sargent (2001) and Klibanoff et al. (2005) as well as on more recent analyses in the context of environmental and resource economics, e.g. Manoussi et al. (2018). An important challenge of the environmental discipline is to provide a framework for the global economy providing the conditions for resilience against major shocks and negative environmental events (Bretschger and Vinogradova 2018). With deep uncertainty one has to generate rules for deep resilience. Including uncertainty is especially important when environmental events do not occur constantly but cause the crossing of tipping points involving large and sudden shifts. Economic modeling needs to increasingly incorporate tipping points and the value of resilience in theory and to generate and use data supporting the empirical validity. The combination of uncertainty and potential irreversible outcomes (e.g., species extinction) is another big challenge for research.
4. **Disruptive development and path dependencies ** Substantial and sometimes disruptive changes in behavioral patterns, economic structure and technologies will be required if net zero GHG emissions and the UN sustainable development goals are to be reached. On the bright side, development may exhibit favorable disruptions. Consumers’ preferences and political pressure coupled with new technology achievements may alter certain sectors in a short period of time. Similar to the communication industry which has completely changed, transportation and heat generation could and mst probably will undergo fundamental changes in the near future. The research challenge here is to provide adequate models predicting and adequately analyzing such important transitions and to highlight resisting forces at the same time. In fact, the change of trajectories in development is often hampered by technological, economic and behavioral lock-ins, resulting in path dependencies and inertia. In such situations, history influences current development through, for example, past investment in R&D, the size of established markets, increasing returns or habits acquired (Aghion et al. 2016; Barnes et al. 2004; Arthur 1989). Behavioral path dependencies affect acceptance and adoption of new technologies, hinder social innovation and might render policies aimed at marginal changes ineffective. They can thus postpone the transition to a low-carbon economy, harm efforts in biodiversity conservation and prolong unsustainable resource use patterns and lifestyles, even if they are welfare enhancing in the long-run (e.g. Acemoglu et al. 2012; Kalkuhl et al. 2012). Inertia and lock-ins may also be policy driven with, for example, political or economics elites trying to block change (Acemoglu and Robinson 2006) or clean energy support schemes fostering new technology lock-ins. Whether disruption or a lock-in emerges depends, for example, on expectations determining the steady state of an economy (Bretschger and Schaefer 2017). This requires nonlinearities e.g. in capital return, generating overlap regions in which the growth path is indeterminate and could be either driven by history or by expectations. The challenge is to add more substantial research into system dynamics and the political economy of change, to gain a better understanding of the different mechanisms responsible for inertia and disruptive change. So far, the role of path dependencies has often been neglected in empirical as well as theoretical analyses (Calel and Dechezlepretre 2016). Also, understanding the triggers or tipping points for disruptive change can help to identify policies that have a big environmental impact with moderate costs in terms of environmental policy.
5. **Behavioral environmental economics ** Traditionally, economics focuses predominantly on the supply side when analyzing potentials and challenges for environmental policies. Preferences of individuals are mostly assumed to be given with economic analysis confining itself to studying the effects of changing incentives and altering constraints. The change and development of preferences over time plays only a comparative minor role for economic research. Also, the follow-up question whether policies should be allowed to tamper with preferences is rarely discussed with nudging being one big exception to this rule (e.g. Strassheim and Beck 2019). While the traditional, supply-side oriented analysis has provided powerful results in positive analysis, it proves to be limited in a field which inherently includes normative conclusions like environmental economics. The path toward sustainable development requires behavioral changes and political actions changing our relationship to the environment. Ultimately, environmental policies have to be decided by the same people overusing the environment in the absence of a policy. In situations where outcomes are inefficient because individuals and political actors follow their own self-interest and ignore external costs and benefits of their actions, it is clearly not sufficient for economists to advocate the implementation of environmental policies. It is crucial to understand under what conditions preferences change and agents support green policies (Casari and Luini 2009). So, the challenge to economic research is to better understand the evolution of green attitudes, the emergence of preferences for a clean environment, and expectations in the case of multiple equilibria (Cerda Planas 2018). The formation and development of preferences is also not independent from cultural, regional and community aspects. Research that ignores heterogeneity among actors or the role of social and group dynamics and only relies on the traditional, isolated analysis of individual preferences is likely to lead to an incomplete understanding of preference dynamics. As the example of discounting shows, the social context has an impact on myopic attitudes and the motivation to undertake sacrifices for a cleaner future (Galor and Özak 2016). Also, attention to behavioral details, that economists might find rather uninteresting from a research perspective, might influence effectiveness of policies tremendously (Duflo 2017). Especially with the natural environment, the choice and guise of policy instruments should take these mechanisms into account.
6. **Institutional analysis of environmental policy** Virtually every contribution to the environmental and resource economics literature culminates in one or several policy conclusions. However, these results are often received with skepticism from industry and public. Therefore, a continuing key challenge for our profession is a thorough understanding of environmental policy institutions, processes and decision-making; this task has become even more important given the enormous scale and global nature of future policies. Research in this area has, however, the advantage of already looking back on a long tradition (see e.g. the body of work by Daniel Bromley, e.g. Bromley 1989). Well-designed institutions support and create incentives to drive development toward a welfare-improving state. Absent, weak, inefficient, or even corrupt governments and institutions are detrimental to successful environmental policy (Pellegrini and Gerlagh 2008; Dasgupta and De Cian 2016) or might lead to detrimental effects of resource wealth (see Badeeb et al. 2017 for an overview of the related literature). To effectively increase social welfare by, for example, conservation of ecological services, one has to design policies in a way that allow implementation under realistic policy conditions (Rodrik 2008). Pure reference to the construct of a social planner is not sufficient. For increasing efficiency in problem solving, the ex-post evaluation of policies has to be expanded and improved. Policy evaluation should not only analyze if regulatory objectives have been reached but also which side-effects arise (OECD 2017). Moreover, the comparison with alternative measures and a continuous international exchange of best practices have to be supported by science. A proactive environmental policy analysis should furthermore include studying vested interests, lobbying, political power, policy communication, and voting behavior. Especially insights from behavioral economics may add to our understanding of a proper design of environmental institutions. On the international level, the adequate institutional design for global environmental policy still poses great challenges. Beyond traditional research fields like international environmental agreements in specific areas like climate change, the multi-dimensionality of the sustainable development goals (SDGs) and potential trade-offs between different goals need to be explored further. This holds especially given the vast differences in income, vulnerability, and resilience between countries, as well as the need for unanimity and voluntary contributions on the UN level. Relating national to international policies has the potential to be especially rewarding in this context given the SDGs relevance for and acceptance in national as well as international politics. Insights from the analysis of institutions in traditional economic sectors (e.g. on the efficiency of capital markets) should be transferred and applied to the global level (e.g. with respect to investment in the world’s natural capital stock).
7. **Equitable use of the environment** We place equity and fairness in dealing with the natural environment on the priority list of our challenges because first and foremost equity is a central requirement for sustainability of development. By definition, sustainable development seeks an equitable treatment across different generations as well as agents living today. We also believe that for successful environmental policies, equity and fairness are crucial complements to the dominant efficiency requirement (Sterner 2011). It is a specific challenge of our field to study equity in an economic context and to demonstrate its importance for sustainability to mainstream economics and the public. The first aspect of the problem is the aforementioned unequal vulnerability of countries to environmental changes such as global warming. If vulnerability is higher in less developed countries, the equity perspective is especially striking. As a matter of fact, most of the climate vulnerable countries have a low average income. Global environmental policy is then motivated not only by efficiency but also by the aim of preventing increasing inequalities (Bretschger 2017a). Global efforts are also indicated to avoid adverse feedback effects of induced inequalities like environmental migration. The second aspect is that acceptance of public policies sharply increases with the perceived fairness of the measure (Pittel and Rübbelke 2011; IPCC 2018). In the past, economists have often underestimated political resistance against efficient environmental protection, which was mostly related to negative impacts on income distribution. Take carbon pricing and emission regulation as a current example. Although evidence from cross-country studies suggests that regressivity of carbon pricing is much less frequent than often assumed in the public (Parry 2015), the perceived distributional impact is often very different (Beck et al. 2016). Therefore the impact of environmental policies on income groups, regions, and countries should be better integrated in our analysis and policy recommendations. Where efficient policies are regressive, economists have to evaluate and propose alternative or complementary policy designs. Benefits and costs need to be disaggregated by group (country) with a special attention on the poorest members of society (countries). Internationally, equity concerns need to be addressed especially in situations where the entire world benefits from the protection of natural capital and ecosystem services in poor countries (e.g., of carbon sinks and biodiversity hubs like tropical rain forests). The experience with the REDD+ process shows the complexity of designing such international approaches to incentivize and enable developing countries to protect these global public goods. More economic analysis is needed on all of the above aspects, giving rise to a rich research agenda in theory and applied work.
8. **Loss of biodiversity and natural capital ** The rate of species extinction today is estimated to be up to 1000 times higher than without human interference (Rockstrom 2009). Human activities impact biodiversity through land use change, pollution, habit fragmentation and the introduction of non-native species but also increasingly through climate change and its interaction with already existing drivers of biodiversity change (IPCC 2002). In view of this, biodiversity conservation has long been a focus of politics. In 1992, the United Nations Convention on Biological Diversity main objectives were stated as ”the conservation of biological diversity, the sustainable use of its components and the fair and equitable sharing of the benefits arising out of the utilization of genetic resources” (UN - United Nations 1992). Yet, although economists have developed conceptual and theoretical frameworks addressing the valuation of biodiversity (Weitzman 1998; Brock and Xepapadeas 2003) and despite data on valuation having become increasingly available (see, e.g. TEEB 2020), Weitzman (2014) points out, that an objective or even widely agreed measure of biodiversity and its value is still missing. The same holds for an underlying theory framework and a comprehensive measure of natural capital that not only includes biodiversity but also its links to regulating services (e.g., pollution abatement, land protection), material provisioning services (e.g., food, energy, materials), and nonmaterial services (e.g., aesthetics, experience, learning, physical and mental health, recreation). How biodiversity and natural capital should be measured, which societal, political and economic values underlie different measures and valuation and how ecological and economical trade-offs should be dealt with are big challenges left for future research. In order to address these issues, not only do we need to develop appropriate assessment methods, but we also need to disclose the theoretical basics of this assessment and which trade-offs go hand in hand with different assessments (Brei et al. 2020; Antoci et al. 2019; Drupp 2018). Completely new issues for the valuation of biodiversity and natural capital arise with the development of new technologies. Take DSI (digital sequence information), for example. DSI are digital images of genetic resources (DNA) that can be stored in databases. This gives rise not only to new challenges regarding their valuation but also about the fair and equitable sharing of the benefits arising out of the utilization of these resources.
9. **Valuing and paying for ecosystem services ** Related to the question of biodiversity valuation is the market and non-market valuation of ecosystem services in general and the adequate design of payment for ecosystem services (PES). Overall, research on ecosystem services valuation has made significant progress in the last decades. Nevertheless, challenges remain even in traditional valuation fields (for example, valuation of non-use or interconnected ecosystems). Other, so far underresearched areas that constitute promising fields for future research are health-related valuation aspects (Bratman et al. 2019) and nonmaterial ecosystem services, such as amenities of landscapes or cultural ecosystem services (Small et al. 2017; James 2015). Also, data availability remains a problem in many valuation areas. Although digitized observation and information systems offer large potentials for previously unknown data access, they also raise a whole slew of new ethical, privacy as well as economic questions, especially in areas like health. While a lot of progress has been made in the valuation of ecosystem services, their impact on decision making still lags behind. One factor contributing to this disconnect are prevalent mismatches between regional and temporal scales of economic, institutional and ecological systems that make valuation and policy design complex (Schirpke et al. 2019). The challenge is to develop combined natural science-economic models that allow better insights into how changes in economic systems lead to changes in the flows of ecosystem services and vice versa (Verburg et al. 2016). This requires a deep understanding of ecological and economic systems as well as other aspects like technologies, regional heterogeneity and system boundaries, i.e. catastrophic events. It also raises classic economic problems, such as choosing an appropriate discount rate and degree of risk aversion. Regarding tools to include ecosystem services in economic decision making, PES are a, by now, well-established (Salzman et al. 2018) and also quite well-researched approach for promoting environmental outcomes. Still, the literature has identified a number of aspects to be addressed in the design of PES to make them more effective as well as efficient and to simultaneously improve social outcomes (Wunder et al. 2018; Chan et al. 2017). A promising area of research rarely addressed are PES to preserve transboundary or global ecosystem services through international payment schemes (for example, in tropical forest preservation). While some work has been done on the conceptual level (e.g. Harstad 2012), the REDD+ process (Maniatis et al. 2019) and the failure of the Yasuni initiative (Sovacool and Scarpaci 2016) show the complexity of such approaches for which a thorough economics analysis is still missing.
10. **Conflicts over natural resources ** Climate change and decarbonization transform regional and global geopolitical landscapes and might give rise to future domestic as well as international conflicts (Mach et al. 2019; Carleton and Hsiang 2016). First, decarbonization changes the role of resources and of resource- and energy-related infrastructures. Climate policies affect the rent allocation between different fossil fuels like, for example, coal and natural gas, but might also change the overall rent level (Kalkuhl and Brecha 2013). Asset stranding can endanger stability in resource (rent) dependent countries. Conflicts may also arise over materials critical to new, low-carbon energy technologies like rare earth elements but also over access to sustainable energy (Goldthau et al. 2019; O’Sullivan et al. 2017). Further research is needed to design policies that are better equipped to reduce the vulnerability of economies to changes in resource availability and resource rents. This opens up challenges for future research, especially as restrictions from very diverse institutional capacities have to be considered to render policies efficient and effective. Second, climate change will affect the ability to meet basic human needs through food, land and water. Sulemanaa et al. (2019) find a positive effect of the occurrence of temperature extremes on conflict incidence. They stress the need for more advanced spatial econometric models to identify effects that are transmitted across space. More research is also needed on the role of institutions and interaction with other phenomena like population dynamics, migration, and environmental degradation. Currently, the role of climate for conflict is still small compared to other causes, many linkages between conflicts and climate change as well as other factors promoting conflict are still uncertain (Mach et al. 2019). The challenge to economic research is to get early insights into the nexus of historical and cultural factors, vested interests, population dynamics and climate change in order to help to prevent resource-related conflicts.
11. **Population development and use of the environment ** Already since antiquity, demographic analysis has been a central topic of human thinking. With the Malthusian predictions of catastrophes caused by population growth, the topic is firmly related to the natural environment and the limits of planet Earth. While limited food production was the dominant topic in the 18th century, the impact of world population on global commons, availability of renewable and exhaustible resources, and ecosystem services have been dominant topics in the last decades. Still, while it is often argued in the public and in natural sciences that world population size should be a concern because of ecological constraints, economics has largely left the topic on the side; the few exceptions (Peretto and Valente 2015) and (Bretschger 2013, 2020) point in a different direction, namely the compatibility of population growth and sustainable development under very general conditions. Current trends of demographic transition show significant signs of population degrowth for leading economies while trends for developing countries vary substantially (UN - United Nations 2019). Population is forecasted to expand especially in Africa, accounting for more than half of the world’s population growth over the coming decades, raising questions about the effect of this population increase on fragile ecosystems, resource use and ultimately the potential for sustainable growth (African Development Bank 2015). Population growth will also promote further urbanization and migration triggered by environmental and resource depletion but also giving rise to new environmental problems (Awumbila 2017). Challenges from population development and environment are thus closely linked to the other research topics highlighted in this article. However, population growth is not exogenously given but determined by economic, social as well as environmental factors. Education and income or economic development have long been established as crucial for fertility (see e.g. the reviews of the literature provided by Kan and Lee 2018; Fox et al. 2019). To integrate these findings into a holistic approach is a mediating challenge for future research. Climate change might affect these channels in different ways, potentially exacerbating global inequality (Casey et al. 2019). However, population development, fertility, and mortality are not only affected by climate change but also by other environmental stresses like air pollution (Conforti et al. 2018). A successful combination of endogenous fertility and mortality with natural resource scarcity, agricultural production, and pollution accumulation as well as capital and knowledge build-up in a comprehensive framework is a respectable challenge for an economic modeller; we suggest that in the future it should be considered by economists more intensively.
12. **Land use and soil degradation** The terrestrial biosphere with its products, functions and ecosystem services is the foundation of human existence, not only for food security but far beyond. Currently, about a quarter of ice-free land area is degraded by human impacts (IPCC 2019). The optimal use of scarce land resources becomes an even more urgent topic in the face of the biodiversity crisis and the onset of climate change. This holds especially as the physical and economic access to sufficient, safe and nutritious food is the basic precondition for human existence. Climate change challenges this access on different levels. On the one hand, climate change increases the pressure on productive land areas (due to extreme weather events such as droughts, floods, forest fires or the shifting of climatic zones). On the other hand, land plays a major role in many climate protection scenarios by reducing emissions from land use and land use change, protecting carbon stocks in soils and ecosystems, and conserving and expanding natural carbon sinks. Also, the capture and storage of CO_2_ through carbon dioxide removal technologies plays an increasing role for reaching the Paris climate goals (IPCC 2018). The induced increase in the demand for the different services from land inevitably implies trade-offs. However, neither the trade-offs nor the potentials for synergic uses are, as of now, comprehensively understood from an economic point of view and thus pose a challenge for future research. While there is a growing literature on negative emission technologies, their costs, potentials and side effects (Fuss et al. 2019 and references within) as well as on the interaction between climate goals and other SGDs on the global level (von Stechow et al. 2016), many research questions still remain to be addressed (Minx et al. 2018). This concerns especially a better understanding of opportunity costs, governance requirements, regional and distributional effects as well as of acceptance and ethical considerations. With respect to land degradation and land use for food production, changing climate and weather conditions as well as regional population pressure may raise the rate of land degradation (Fezzi and Bateman 2015), hurting food security and calling for preservation policies (Brausmann and Bretschger 2018). The overuse of ecosystems like forests and water, which protect and complement land, can accelerate the risk of adverse shocks and thus lower soil fertility, which reveals the close link between the different research subjects. However, much of the agricultural research in this field is still quite distant from mainstream environmental economics which can harm research productivity substantially. It remains a challenge to integrate agricultural and environmental research better, for example by bringing together food production, population, and the environment into a macrodynamic framework (Lanz et al. 2017).
13. **Environmental migration** Migration in times of climate change is an extraordinarily complex, multicausal and controversial challenge (Adger et al. 2014). Heatwaves, droughts, hurricanes, and rising sea levels are likely to motivate or even force a growing number of people to leave their homes moving to presumably safer places. Climate-related migration can take a variety of different forms (Warner 2011) from voluntary to involuntary, from short- to long-distance and from temporary to permanent. Migration decisions are usually based on different motives and personal circumstances (climatically, politically, economically, socially), leading to heterogeneous reactions to climate events and making it often problematic to identify and delineate climate-induced migration. Due to these and other methodological difficulties and the small number of studies so far, no globally reliable forecasts for climate induced migration exist (WBGU - German Advisory Council on Global Change 2018a, b). At present, the forecasted magnitude of the phenomenon ranges from 25 million up to 1 billion people by 2050 (Ionesco et al. 2017). Much of this migration can be expected to take place within countries, for example, from rural to urban areas or from drylands to coastal zones (Henderson et al. 2014) with environmental migration being one possible adaptation and survivor strategy in the face of climate change (Millock 2015). Given the uncertainty in future migration projections, the challenge is to improve migration models (Cattaneo et al. 2019) which includes a better understanding and integration of the microfoundation of agents’ migration decisions. Migration, and especially mass-migration, can have a profound impact on the environment of the new as well as the old settlement location and on their economic structure. Labor and commodities markets will be affected the most, with challenges arising also for education and health systems, government budgets and public spending. By affecting public institutions and the skill-mix of the labor force, migration alters economic development both in the sending and in the receiving countries or regions. More research is needed on these impacts. The influx of environmental migrants to new settlement locations may also trigger hostile attitudes and lead to clashes and even armed conflicts. The migrants may be perceived as rivals for scarce resources (land, clean water) or jobs. The situation may be aggravated by lack of political stability and poor-quality political institutions. Dealing with these aspects gives rise to new challenges in environment and resource economics. Traditional analysis of economic costs and benefits of migration have to be complemented by behavioral economic and political economy analyses.
14. **Urbanization as a key for environmental development** In the last 70 years, the urban population has increased fivefold with more than half of the world’s population living in cities today and forecasts projecting the share of urban population to rise to almost 70% in 2050 (UN - United Nations 2018). Cities are responsible for about 70% of the world energy use and global CO$_{2}$-emissions (Seto et al. 2014) and ecological footprints are positively correlated to the degree of urbanization (WBGU - German Advisory Council on Global Change 2016). In 2014, about 880 million people were living in slums (UN - United Nations 2016) elucidating the problems to make urban development environmentally as well as economically and socially sustainable. The speed of urbanization is projected to be the fastest in low and middle income countries, especially in Africa and Asia (UN - United Nations 2018), leading to new challenges for the provision of infrastructure, housing, energy supply, transport and even health care. Climate change can be expected to not only foster urbanization trends (Henderson et al. 2017) but also increase the magnitude of urbanization-related challenges. Urban areas are often located close to the coast or rivers basins, making them susceptible to rising sea levels and impacts of extreme weather events. Risks can be expected to be higher for poor households due to settlement in less safe areas and poorer housing (Barata et al. 2011), potentially perpetuating existing inequalities. On the other hand, cities might offer more efficient adaptation potentials. To date the consequences of climate change for cities and urbanization are still to be determined in detail but depend heavily on factors like location, size and level of development as well as governance capacities. Making cities, their population and their infrastructure resilient to climate change will be decisive for future development. The main challenge here is to better connect the research fields of environmental and urban economics to understand the drivers and dynamic effects of climate change on urbanization and resulting economic development, on adaptation costs and benefits and on the role of institutions. Insights from regional, political and behavioral economics can help shape effective governance to enhance resilience of cities to climate change.
15. **Health and epidemiological environment** Environmental degradation can have profound implications for human health. These implications lead to direct as well as indirect challenges for economic decision making, economic development and thus economic research. While many of these challenges might not be new per se, they can be severely exacerbated by, for example, climate change. Economic implications of long-term increases in vector-borne diseases and heat stress as well as pandemics like the COVID-19 and ozone formation still remain to be analyzed in depth, as do the costs and benefits of adaptation measures dedicated to mitigating these effects (Mendelsohn 2012). Climate change also affects human health indirectly through impacts on economic development, land use, and biodiversity - and vice versa. Failed emission reductions and bad environmental management especially impact developing countries negatively through direct effects on health but also through health effects of delayed poverty reduction (Fankhauser and Stern 2020). Exposure to diseases or epidemics can increase the risk of civil conflicts and violence (Cervellati et al. 2016, 2018). While research has addressed effects of life-expectancy, diseases and premature mortality on long-run economic development (e.g. Ebenstein et al. 2015; Acemoglu and Johnson 2007), a thorough analysis of the climate-health-development nexus is still missing. Overall, most research carried out on the interaction between environment, climate and human health has focused on physical health and mortality. The effects of air pollution from the burning of fossil fuels or agriculture on premature deaths, cardiac conditions and respiratory diseases, for example, received not only renewed interest in the wake of recent scandals (see e.g. Alexander and Schwandt 2019) but have been an active field of research for a number of years (Schlenker and Walker 2016; Tschofen et al. 2019). Mental health implications like stress, anxiety or depression on the other hand have received much less attention although, for example, Chen et al. (2018) in a study on air pollution in China estimate these effects to be on a similar scale to costs arising from impacts on physical health. Also, Danzer and Danzer (2016) find substantial effects of a large energy-related disaster (the Chernobyl catastrophe) on subjective well-being and mental health. Economic research should take up the challenge and put more effort into the economic evaluation of mental health related effects of climate change and environmental degradation in general. Potential to analyze these and other health-related questions have risen substantially in the last years, method-wise as well as topical, with new large data sets becoming available. Big data from insurance companies, satellite imagery on pollution dispersion and effects of draughts, for example, can provide new insights into the dynamics between environmental changes and health. But digital technologies themselves also generate new research questions addressing, for example, risks, costs and benefits of these new technologies.
16. **Carbon exposure and green finance ** The impact of climate change and of climate policy on the financial system is a topic of increasing public concern. The transition to a low-carbon economy poses a lot of challenges not only from physical risks and damages but also from transition risks. These accrue in such different areas as climate-related policy making, altered market behavior, changes in international trade patterns, technology development, and consumer behavior. To support a safe and gradual transition to a low-carbon economy, the financial sector needs to evaluate and eventually address the new risks associated with climate change and decarbonization in an efficient manner. There is widespread concern that financial markets currently lack sufficient information about the carbon exposure of assets, resulting in risks from climate change and climate policy for investments (Karydas and Xepapadeas 2018). If not anticipated by the markets, climate shocks also cause asset stranding, i.e. unanticipated and premature capital write-offs, downward revaluations, and conversion of assets to liabilities (Rozenberg et al. 2020; Bretschger and Soretz 2018). The same holds true for climate policies which are not or cannot be correctly anticipated by investors (Dietz et al. 2016; Stolbova et al. 2018; Sen and von Schickfus 2020). The growing awareness of these risks is reflected in the attention that policy makers have devoted to the development of transparency improving information systems and indicators in recent years. However, challenges related the design of these systems and indicators, e.g. with respect to an accurate and encompassing risk assessment, still remain. The importance of addressing these challenges is excerbated by prevalent network effects and counterparty risks that transmit climate-induced financial shocks from individual firms to the broad public holding their capital in stocks of fossil-fuel-related firms, investment funds, and pension funds, which all could suffer from stranded assets (Battiston et al. 2017). Divestment campaigns, shareholder engagement, and mandatory disclosure of climate-relevant financial information by companies and investors warrant further theoretical and empirical analysis. Also, a better understanding of the economics behind financing instruments like green bonds is only recently emerging (Agliardi and Agliardi 2019). Despite some early studies there is a knowledge gap with respect to the extent of climate and policy risks for central banks and regarding the potential significance of different channels connecting the risks in the real economy with monetary policy. Given the environmental and international policy perspective of the climate problem, the specific contribution of the financial sector and the central banks in the architecture of global climate policy has to be subject to further investigation.
17. **Energy system transformation ** The transition from a fossil-based to a green economy is needed to combat climate change but requires a thorough transformation of energy systems (Pommeret and Schubert 2019) in developed as well as in developing countries. In industrialized countries, challenges arise from the structural transformation of highly complex energy systems and their linkage with other economic sectors. While one hundred years ago, it was the rapid dissemination of fossil-based industrial processes, transportation, and heating that resulted in wide-spread sectoral change, similar adjustments can be expected with the increasing importance of electricity for decarbonization. However, changing the use of energy technologies in practice involves decisions on different levels and constitutes a highly nonlinear process. Future power generation in many countries will increasingly rely on renewable energies like wind and solar energy. To offset intermittent power generation, more and better storage capacities of batteries or pumped hydropower will be needed (Ambec and Crampes 2019). Synthetic fuels, heat pumps, fuel cells and e-mobility will increasingly use electricity to replace fossil fuels not only in the power sector but also in traffic and heat generation. While the adoption of renewable technologies like wind and solar was often much faster than predicted in the past, the critical mass of market penetration has still to be reached in other areas to benefit from potential scale effects and cost decreases. Shape and speed of the energy transition are, however, highly dependent on a political process which is hard to predict for market participants. Policy and ecological risks, together with the long-run character of the energy and related infrastructure investments, pose a big challenge for research and practice. In this context, it is especially the economic potential of green hydrogen and/or synthetic fuels that is controversially discussed at present. As production costs are expected to fall (Glenk and Reichelstein 2019), interest in hydrogen is increasing sharply (IEA 2019) and new research questions arise. For developing countries, clean and decentralized renewable energy technologies offer big potentials for electrification and economic development. However, despite the potential for decarbonization and the reduction of other externalities and health hazards and despite the fact that more than 90% of the annual increase in power generation comes from emerging economies, research on the development and adoption of clean energy technologies still focuses mainly on the developed world. More research on the barriers and challenges for adoption in developing countries is needed, including sustainable financing, institutional framing and the design of regionally tailored policies.
18. **Sustainability perspective on digitalization** Digitalization and artificial intelligence are often seen as opportunities for enhancing the efficiency of energy and resource use. They offer new opportunities for circular economy, agriculture, monitoring of ecosystems and biodiversity, sustainable finance and decarbonization (see WBGU 2019 and literature within). However, they may also accelerate energy and resource use, increase inequality between regions and income groups and endanger sustainable development. Digitalization offers new access to markets, impacts market forms and shapes consumer behavior all of which can have extensive implications for the ecological, social and economic dimensions of sustainable development. Digitalization is a cross-cutting theme that reaches across spatial scales (from regional development to globalization) as well as temporal scales (from short-run impacts on energy systems to long-run adaptation to climate change). So far, the potentials and challenges for sustainable development that are associated with digital technologies have mostly been addressed outside of environmental and resource economics. The focus has been on topics such as data security and privacy or, for example, on the implications of the ”fourth industrial revolution” on employment and labor markets. Costs and benefits of digitization, the design and effectiveness of policies in industrialized as well as developing countries have garnered much less attention in the context of environmental, resource, energy and climate economics. Also, impacts of digitization on the behavior of economic agents resulting in, for example, rebound effects or changes in consumption patterns and environmental awareness, have not been addressed comprehensively (Gossar 2015). In all of these areas, our limited knowledge base creates opportunities and challenges for future research in the field. But, digitalization not only creates new research questions, it also provides new means to answer them. It has led to new developments in data science, big data analysis, machine learning and artificial intelligence that allow new insights into, for example, material flows, emission patterns and technology diffusion as well as the optimal design, implementation and effectiveness of regulation (Fowlie et al. 2019; Weersink et al. 2018; Graziano and Gillingham 2015).
19. **Quantitative analysis of environmental use** Recently, there has been a significant shift in the empirical methods used in economics from traditional regression analysis to random assignment and quasi-experiments. Arguably this can improve the capturing of causal relationships and reduce the biases of traditional study designs. In environmental economics, experimental and quasi-experimental approaches have been applied mainly for capturing individuals’ or firms’ decisions on the use of land, water, resources, and energy (e.g. Allcott 2011; Duflo et al. 2013; Deschenes et al. 2017). Wider applications of these rigorous methods in environmental economics and well-suited empirical designs are desirable but certainly challenging e.g. when assessing aggregate environmental costs from climate change or biodiversity loss. An important but underrated field in applied environmental economics is the ex-post empirical assessment of environmental policies. The challenge is not only to identify environmental externalities, causalities, and impact intensities but also to provide an accurate valuation of the cost of policies, because they vary widely especially in environmental economics. The traditional empirical methods remain to be important and are not simply replaced. The same holds true for empirical designs in a time, cross-country, or panel structure. The increasing availability of large or very large datasets with observations varying widely across time and space offers a different set of options to provide evidence on the impact of environmental damages or policies to abate them (e.g. Currie and Walker 2011; Martin et al. 2014; Zhang et al. 2018). Fast-growing computational power and machine learning provide even more avenues for fruitful applications in environmental economics (see e.g. Abrell et al. 2019) but the challenge to use computer power wisely and to derive results which are sufficiently robust remains demanding .
20. **Structural assessment modelling and modelling transparency** In an effort to better understand the ramifications of political decisions and technological developments on climate change, energy supply and resource extraction (to name but a few examples), increasingly sophisticated numerical models have been developed in recent decades. It is evident that quantitative economics analysis is important for policy advice. Yet despite their complexity, these models usually still adopt some very simplifying and sometimes ad-hoc assumptions. In particular assumptions used in integrated valuation models have come under heavy criticism in recent years (Stern 2013; Pindyck 2013). Simplifications concern market structures and market failures, the integration of risk and uncertainty as well as societal, institutional and cultural detail. Also, manifestations of climate change and damages come at very different regional and temporal scales, making a truly integrated assessment of the climate-ecosystem-economy nexus next to impossible. We see it as a major challenge for future research to provide more accurate foundations for integrated assessment models. While simplifications are needed to reduce computational complexity, they raise the question to which extent the results obtained render reliable insights into future developments. Asking for models that are detailed in every dimension and can answer every question resembles of course the search for the holy grail. However, the need for a better understanding of the model dynamics has already led to the development of a new generation of models which have a stronger foundation in theory (Golosov et al. 2014, Bretschger and Karydas 2019). A better understanding of the limits of models and of the questions specific models can and cannot address is still needed as well as transparency in model development. More applied studies, assessments of global environmental trends under different economic assumptions often use ”scenarios” to describe future trajectories. The scenarios are mostly based on expert opinion and do not rely on estimates about the likelihood that such a trajectory will occur. It is also critical that the economics behind the scenarios is often neglected. Prominently, per capita income can be projected using endogenous growth theory, while population development can be evaluated using state-of-the-art theories on fertility and morbidity.

## Conclusions

This article set out to highlight a number of challenges that are highly relevant for future research in the field of environmental and resource economics. The focus was mainly, although not exclusively, on topical issues. We only briefly touched upon on some methodological advancements that might have the power to further parts of our field. Big data, machine learning and artificial intelligence hold high promise in this regard but their limits and potentials for environment, climate and resource economics have yet to be fully understood.

It should have become clear, that a number of the challenges presented can only be addressed adequately by interdisciplinary research teams with relevant disciplines ranging from climate science, (computer) engineering, sociology, virology to soil sciences. In many cases, economists’ analysis and the derivation of sound policy recommendations require the knowledge available in these fields. However, such research cooperations are by no means one-way streets: Other disciplines need the input of economists in order to assess future development scenarios and implementability of solutions. The knowledge and data required for economics analysis does not always exist yet, but interdisciplinary cooperation can help to identify and close these gaps. Overall, the less economists have already worked on specific challenges, the harder it is to assess best research strategies and the potential for success. Take the digitization-sustainable-development-nexus as an example: best research strategies and success are extremely difficult to predict as not only is the related economics research still in its infancy but also the field itself is extremely dynamic.

As already pointed out in the beginning: We are aware that our selection is bound to create discontent and disagreement. Having said this, it should also be stated that we expect some of our challenges to be more or less universally agreed upon. This holds especially for the broader topics: for example, how to accomplish deep decarbonization; how to deal with risk and uncertainty; or how to assess the role of disruptive development. One reason for this lies in the encompassing nature of these topics. They are relevant for many of the other fields that we have pointed out: For behavioral analyses, the capacity to deal with disruptive change in the face of risk and uncertainty are essential. Loss of biodiversity and natural capital, land degradation, conflicts over resources and migration are exacerbated by climate change. The potential of digitization for sustainable development constitutes disruptive change in itself. Yet, all of these fields are not merely subfields of the more overarching themes, they raise important research questions in their own right.

Nevertheless, it is to be expected that it will be the more specific fields over which disagreement will arise: Are ‘land use and soil degradation’ more important than ‘fisheries’? Is the ‘institutional analysis of environmental policies’ of higher relevance than the ‘development of alternative welfare concepts’ (to pick out some random examples). Of course, there are more fields that could have been included and also, of course, there is no objective criterion for the inclusion or exclusion of fields. The selection of the challenges is based on the analysis and criteria presented in the first section but it is ultimately ours; we are happy if this paper contributes to a lively and constructive discussion about the future of our field.
